# A study for the genotoxicity assessment of substances containing probiotic candidates in the in vitro TK6 cell micronucleus test: Influence of low pH conditions and antibiotic supplementation on the test results

**DOI:** 10.1186/s41021-024-00320-w

**Published:** 2024-12-18

**Authors:** Yohei Fujiishi, Wakako Ohyama, Emiko Okada

**Affiliations:** Yakult Central Institute, Yakult Honsha Co., Ltd., 5-11 Izumi, Kunitachi-Shi, Tokyo 186-8650 Japan

**Keywords:** TK6 cell, Micronucleus test, Medium pH, Antibiotics, Probiotics

## Abstract

**Background:**

When assessing the genotoxicity of substances containing probiotic candidates, such as lactic acid-producing bacteria, using the in vitro micronucleus test (MNT), bacterial growth in the test medium may reduce the pH of the medium. The low medium pH is known to induce cytotoxicity and false-positive results. In the TK6 cell system, it is difficult to completely remove the bacteria from the medium by washing post-treatment, leading to bacterial growth during the recovery period in the short-term treatment. In the present study, the low pH range yielding false positives in the TK6 cell MNT was investigated using media supplemented with acetic, lactic, or formic acids, which are non-genotoxic bacterial metabolites. Additionally, to suppress the bacterial growth during the recovery period using antibiotics, i.e., penicillin/streptomycin (P/S), gentamicin sulfate (GM), and amphotericin B (AP), the maximum applicable concentrations of them that did not affect TK6 cell growth or micronucleus induction were determined. Then, we conducted an MNT using a substance containing live lactic acid-producing bacteria to verify the effectiveness of the antibiotics.

**Results:**

Acetic, lactic, and formic acids induced micronuclei in TK6 cells (false positive) at an initial pH of ≤ 6.2 and ≤ 6.0 in 3 h treatment with and without S9 mix, respectively, and of ≤ 6.7 in the continuous treatment. Media supplemented with P/S, GM, and AP did not affect TK6 cell growth or micronucleated cell frequencies in the negative and positive controls ≤ 400 unit/mL-400 µg/mL, ≤ 250, and ≤ 20 µg/mL, respectively. In an MNT with fermented milk containing live lactic acid-producing bacteria, supplementation with P/S or GM to media for the recovery cultures suppressed the bacterial growth, decreasing pH, and cytotoxicity.

**Conclusion:**

This study revealed the low pH ranges yielding false positives in the TK6 cell MNT under short-term and continuous treatment conditions. These values will serve as references for interpreting the biological relevance of results. Under short-term treatment, optimal antibiotic supplementation in recovery cultures suppressed bacterial growth in the test substance and prevented the decrease in pH that could yield false positives. This approach might be useful for evaluating the genotoxicity of test substances containing probiotic candidates using the MNT.

## Introduction

In response to the animal welfare movement, i.e., the ‘3Rs’ (animal use reduction, replacement, and refinement), the importance of evaluation in in vitro tests without the use of animals is increasing. When assessing the genotoxicity of substances containing probiotic candidates, such as fermented products intended for oral ingestion, using the test substance without killing the bacteria and with their metabolites is preferable. However, in vitro tests with such substances are challenging because the tests require aseptic manipulation and sterile test substances. In the micronucleus test (MNT) using cultured mammalian cells, bacterial growth in the test medium may reduce the pH of the medium, inhibit cell growth (induce cytotoxicity), and produce false positives during treatment and recovery periods. Completely removing the bacteria contained in the test substance by washing after treatment is a challenge of bacterial growth during the recovery period, particularly in non-adherent cell systems. We observed growth from the remaining bacteria in the recovery cultures, which reduces the final pH of the medium in the MNT using the human lymphoblastoid TK6 cell line.

Chromosomal aberration tests using cultured mammalian cells revealed that chromosomal aberrations (false-positive responses) were induced under low pH conditions [1–4]. Furthermore, the clastogenic response to a low pH varied considerably among cell lines [2, 5–8]. As for MNT using TK6 cells, there are no reports on the effect of low medium pH on micronucleus (MN) induction. In this study, TK6 cell MNTs were conducted using low pH conditions and culture media supplemented with organic acids (acetic, lactic, or formic acids), which are non-genotoxic bacterial metabolites [7, 9] to investigate the pH ranges that yielded false-positive responses in short-term and continuous treatments (Experiment 1).

Kawanishi et al. [10] revealed MN induction in Chinese hamster ovary (CHO) cells by infection with colibactin-producing live *Escherichia coli* (*E. coli*) for 4 h without S9 mix in MNT. They used media supplemented with gentamicin 200 µg/mL during the recovery period post-treatment. Regarding the TK6 cell MNT, there are no reports studying live bacteria-containing test substances. In the present study, we first investigated the maximum applicable concentration of several antibiotics that did not affect cell proliferation or micronucleated cell frequencies when TK6 cells were treated with negative and positive control substances for 3 h followed by recovery culturing for 21 h (Experiment 2). Penicillin/streptomycin (P/S) and gentamicin sulfate (GM) both act on gram-positive and -negative bacteria, and amphotericin B (AP), which acts on yeast, were used as antibiotics in the study.

Finally, we conducted an MNT using fermented milk containing live lactic acid-producing bacteria as the test substance to verify the effects of antibiotics on bacterial growth suppression in recovery cultures (Experiment 3).

## Materials and methods

### Cells and culture conditions

The human lymphoblastoid TK6 cell line (JCRB Cell Bank, National Institutes of Biomedical Innovation, Health and Nutrition, Osaka, Japan) was used. The modal chromosome number of the cells was 47, and the doubling time was 13.4 h. The cells were confirmed to be mycoplasma-free. This cell line was selected because it is recommended according to the Organization for Economic Co-operation and Development Test Guideline (OECD TG) 487 [4], is widely used for in vitro MNTs, and has sufficient historical control data.

Cells were maintained in RPMI 1640, supplemented with 10% (v/v) heat-inactivated horse serum, and 200 µg/mL pyruvate solution (All from Thermo Fisher Scientific Inc., Waltham, MA, USA). Cells were cultured at 37 ℃ in a humidified atmosphere of 5% CO_2_ (Thermo Fisher Scientific Inc.). A cell suspension was prepared in fresh culture medium at a density of 3 × 10^5^ cells/mL and seeded into 24-well plates (Thermo Fisher Scientific Inc.) at a volume of 500 µL to achieve 1.5 × 10^5^ cells/mL (1000 µL in total per well with treatment solution) in each experiment.

### Chemicals

Acetic, lactic, and formic acids (FUJIFILM Wako Pure Chemical Corp., Osaka, Japan) were diluted with distilled water to 2 mol/L on the day of use.

The P/S solution (Thermo Fisher Scientific Inc.) contained 10,000 U/mL penicillin and 10,000 µg/mL streptomycin (hereafter referred to as U-µg/mL). GM solution (FUJIFILM Wako Pure Chemicals Corp.) contained 50 mg/mL GM, and AP solution (FUJIFILM Wako Pure Chemicals Corp.) contained 250 µg/mL AP. Each solution was added to a fresh medium to obtain the required concentration.

Saline (JP, Otsuka Pharmaceutical Factory, Inc., Tokushima Japan) was used as a negative control and added to each well for negative control group at 100 µL in Experiment 2 and 10 µL in Experiment 3. In Experiments 2 and 3, mitomycin C (MMC, FUJIFILM Wako Pure Chemicals Corp.) was used for positive controls for the treatment without metabolic activation, and cyclophosphamide monohydrate (CP, FUJIFILM Wako Pure Chemicals Corp.) was used for the treatment with metabolic activation [4]. Frozen stock solutions (2 µg/mL MMC and 25 µg/mL CP) were thawed immediately before use and added to each well for each positive control group at a volume of 100 µg/mL (0.2 µg/mL MMC and 2.5 µg/mL CP at the final concentration), as significant increases were observed in MN frequencies at these concentrations. Herein, the historical negative and positive control ranges were as follows: negative control ranges, 0.00–1.01% (without S9 mix in short-term treatment); 0.03–0.91% (with S9 mix in short-term treatment); 0.07–0.80% (continuous treatment); positive control ranges, 1.75–5.12% (without S9 mix in short-term treatment); and 1.08–3.20% (with S9 mix in short-term treatment).

The frozen S9 mix (Ieda Trading Corporation, Tokyo, Japan) for the chromosomal aberration test comprised 1.05 mL of a S9 fraction and 2.45 mL of a co-factor solution thawed and thoroughly mixed before use. The S9 fraction was derived from the livers of male Sprague–Dawley rats treated with phenobarbital and 5, 6-benzoflavone. The composition of the S9 mix (1 mL) was as follows: 0.7 mL water, 0.3 mL S9, 5 µmol MgCl_2_, 33 µmol KCl, 5 µmol glucose-6-phosphate, 4 µmol NADP, and 4 µmol 4-(2-hydroxyethyl)−1-piperazineethanesulfonic acid buffer (pH 7.2). The S9 mix (65 µL) was added to each well for the control and treatment groups when the cells were treated in a metabolic activation system. The resulting S9 concentration was approximately 2%.

### Experiment 1. Effect of low pH on cytotoxicity and MN induction

In preliminary studies, cytotoxicity was observed as the pH of the medium decreased, with the index “relative increase in cell count (RICC)” below 0 when the initial pH was approximately 5.7 (short-term treatment without S9 mix), 5.5 (short-term treatment with S9 mix), and 6.3 (continuous treatment). Moreover, when the initial pH was below 6.9, the final pH (at the end of the treatment) did not recover to that of the untreated condition.

**Fig. 1:**
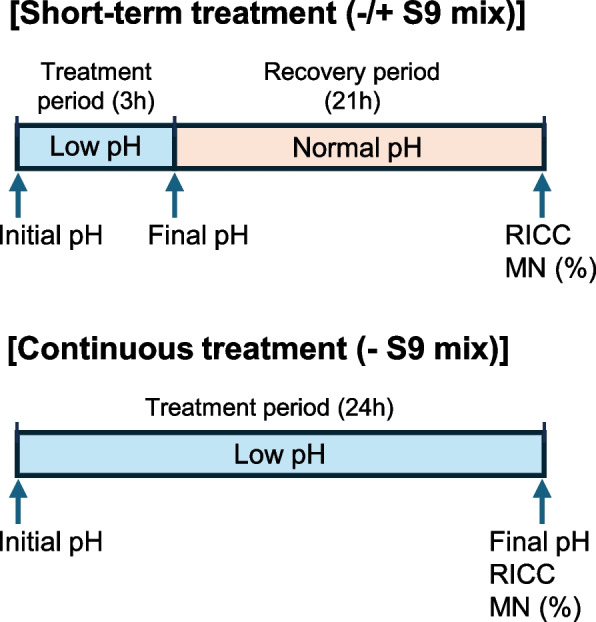
The design of Experiment 1. In short-term treatment, cells were treated with low pH (initial pH 6.6–5.2) for 3 h in the absence or presence of S9 mix, followed by recovery culturing for 21 h using normal media (approximately pH 7.7). In continuous treatment, cells were treated with low pH (initial pH 6.8–6.1) for 24 h without S9 mix. Then, the pH of the medium was measured at the start and end of treatment (3 and 24 h after treatment in the short-term and continuous treatments, respectively). RICC and MN frequencies were determined 24 h post-treatment initiation

Thus, five pH groups of pH ranging from 6.6 to 5.2 (short-term treatment) and from 6.8 to 6.1 (continuous treatment) of the media were set at the start of treatment in the main tests, which were repeated twice. The experimental design is illustrated in Fig. 1. In short-term treatment without S9 mix and in continuous treatment, a 500-µL aliquot of culture medium containing either organic acid (2 mol/L solution, ranging from 6–15 µL) was added to each well, and then a 500-µL aliquot of the cell suspension was added to achieve a density of 1.5 × 10^5^ cells/mL, and achieve a volume of 1 mL per well (2 wells/group), then the wells were mixed gently by shaking the plate. In short-term treatment with S9 mix, a 435-µL aliquot of culture medium containing either organic acid (2 mol/L solution, ranging from 5–12 µL) and a 65-µL aliquot of the S9 mix was added to each well, and the cell suspension was added as described above. Under each treatment condition, the untreated group (approximately pH 7.7) was used as control (two wells/group).

The cells were incubated at 37 ℃ in a humidified atmosphere of 5% CO_2_ for 3 and 24 h for the short-term and continuous treatments, respectively. For short-term treatment, the plates were centrifuged at 300 × *g* for 5 min at room temperature after 3 h of treatment, and an 800-µL aliquot of the supernatant was gently removed from each well. An 800-µL aliquot of fresh medium was added to resuspend and wash the cells, this process was triplicated. The cells were incubated for approximately 21 h (recovery culture) as described above.

The pH of the medium was measured using a pH meter (Horiba pH-11B, Kyoto, Japan) at the start and end of the short-term and continuous treatments (3 and 24 h after treatment), respectively.

The RICC and frequency of cells with micronuclei (MN frequency) were determined 24 h after the start of treatment as described in sections of “Cytotoxicity determination” and “Preparation of MN specimens and MN analyses”, and the mean of two wells/group was calculated.

The result was considered positive when the mean MN frequency in the treated group significantly increased or exceeded the upper limit of the historical negative control range (see the “Chemicals” section) in our laboratory compared with that in the control group.

### Experiment 2. Investigation of applicable antibiotic concentration ranges for recovery culture

In preliminary studies, we examined the cytotoxicity of supplementation with each antibiotic to the medium for recovery cultures in short-term treatment without S9 mix and determined the optimal applicable antibiotic concentration at which RICC was approximately 80% at the end of the recovery period. The P/S, GM, and AP concentrations were 800 U-µg/mL, 1000 µg/mL, and 20 µg/mL, respectively. The lowest concentrations were set to those generally used to maintain cultured cells without contamination [11, 12].

**Fig. 2:**
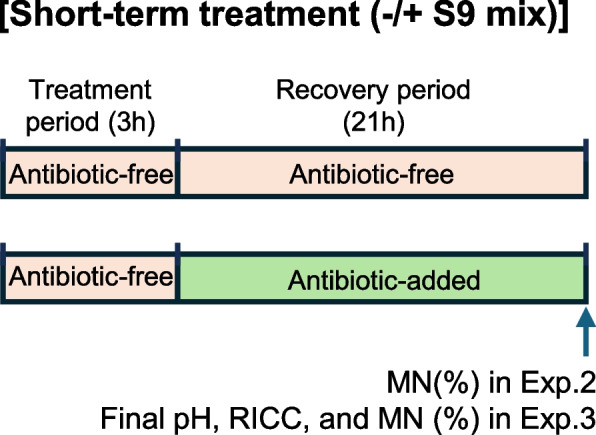
The design of Experiments 2 and 3. Cells were treated with saline (Neg.; negative control) or positive controls (Pos.; MMC or CP) in Experiment 2 and sample A, Neg., or Pos. in Experiment 3 for 3 h using antibiotic-free medium in the absence or presence of S9 mix, followed by recovery culturing for 21 h using media with or without antibiotics. At the end of the recovery period, the MN frequencies were determined in Experiment 2, and the pH of the medium, RICC, and MN frequencies in Experiment 3

To investigate the applicable antibiotic concentration ranges, we prepared a medium for the recovery culture containing antibiotics at the following concentrations: P/S, 100, 200, 400, and 800 U-µg/mL; GM, 50, 100, 250, 500, and 1000 µg/mL; AP, 2.5, 5, 10, and 20 µg/mL. Subsequently, we prepared an antibiotic-free medium and performed the MNT twice. The experimental design is illustrated in Fig. 2. Cells were treated with negative (saline) and positive (MMC) control substances for 3 h in an antibiotic-free medium without S9 mix. After treatment, the cells were washed to remove negative and positive control substances using the antibiotic-added media prepared as described above. The cells were incubated in the same medium for a recovery period of 21 h. The procedures for washing cells, media renewal, and cell incubation were the same as those described in Experiment 1. Specimens for MN analyses were prepared using the method described in “Preparation of MN specimens and MN analyses” section, and 2,000 cells per group (1,000 cells/well) were scored to determine the MN frequency.

Subsequently, in the presence of S9 mix, the cells were treated with saline (a negative control substance) or CP (a positive control substance) for 3 h using the antibiotic-free medium. The subsequent procedure was the same as that described above. The following concentrations of antibiotics were used in the presence of S9 mix: P/S, 100, 200, and 400 U-µg/mL; GM, 50, 100, and 250 µg/mL; AP, 2.5, 5, 10, and 20 µg/mL, because the media containing 800 U- µg/mL P/S and 500 and 1,000 µg/mL GM suppressed the MMC-induced increase in micronucleated cells in the absence of S9 mix. The MN frequencies (mean of two wells/group) were determined.

The concentrations were considered appropriate for application to recovery culture media when the following conditions were met: the mean MN frequencies of the two tests in the negative and positive control groups were comparable between those using media with and without antibiotics, and the values in the positive control groups exceeded the mean of the historical positive control data in our laboratory.

### Experiment 3. Verification of the effect of antibiotics on MNT results using sample A containing live lactic acid-producing bacteria

Sample A was fermented milk containing two live lactic acid-producing bacteria. The bacterial density in milk was > 10^8^ CFU/mL per bacterium, and bacterial growth was observed in the medium cultured with TK6 cells.

We conducted an MNT using TK6 cells treated with sample A, saline (negative control), or positive controls (MMC or CP) for 3 h in antibiotic-free media in the absence or presence of S9 mix, followed by recovery culturing for 21 h in media with or without antibiotics. The experimental design is illustrated in Fig. 2. The volume of sample A and saline was 10 µL/mL/well. The OECD TG 487 for in vitro mammalian cell MNT [4] specifies a maximum concentration of 2 µL/mL for liquid test samples. However, when the test substance is composed of biological materials, the maximum concentration should be higher than that to increase the concentration of each component. Thus, the treatment concentration was set to 10 µL/mL because sample A was composed of biological materials. The bacteria were gram-positive; therefore, P/S and GM were selected. In a preliminary study using media supplemented with 100 and 400 U-µg/mL P/S along with 50 and 250 µg/mL GM during the recovery period post-treatment with sample A, the RICC in the group treated with media supplemented with 250 µg/mL GM decreased to 67%, whereas it was > 80% in the other groups. Therefore, we used media supplemented with 100 and 400 U-µg/mL P/S and 50 µg/mL GM in the main test, and determined that the final pH of the medium (end of the recovery period), RICC, and MN frequencies (mean of two wells/group). The initial pH (at the start of the treatment with sample A) was not measured in the main test because the pH of the medium did not change immediately after the addition of sample A in a preliminary study.

### Cytotoxicity determination

The RICC was calculated per well from the cell seeding density at the start of the treatment and cell density at the end of the culture (end of the recovery period for short-term treatment) using the following formula.

$$\text{RICC} = \frac{\text{Increase in cell density in the treated cultures }(\text{final} - \text{starting})}{\text{Increase in cell density in control cultures }(\text{final} - \text{starting})} \times 100 (\%)$$

The mean of two wells/group was calculated.

### Preparation of MN specimens and MN analyses

The plates were centrifuged at 300 × *g* for 5 min at room temperature, and approximately 800 µL of supernatant was removed from each well. A 1 mL aliquot of 0.09 mol/L potassium chloride solution was added to each well (0.075 mol/L final concentration) for hypotonic treatment. The cells were gently suspended, incubated at 37 ℃ for 10 min, and transferred to a tube containing 0.2 mL of ice-cold Carnoy’s fixative solution-1 (ethanol/acetic acid, 3/1, v/v). After mixing gently, the cells were fixed twice in 1 mL ice-cold Carnoy’s fixative solution-1. The cells were fixed in ice-cold Carnoy’s fixative solution-2 (ethanol/acetic acid, 97/3, v/v) and placed in a freezer overnight. Finally, fixed cells were dropped onto two spots on each slide and allowed to dry.

All slides were coded before microscopic observation. Immediately before observation, cells were stained with a 0.02 mg/mL acridine orange aqueous solution (FUJIFILM Wako Pure Chemicals Corp.) and observed under a fluorescence microscope (600 × magnification) with blue excitation (490 nm). Subsequently, 2,000 cells per group (1,000 cells/well) were scored to determine the MN frequency, and the mean of two wells/group was calculated.

### Statistical analyses

Statistical analyses of MN frequencies between the treated and control groups were performed using Fisher's exact test at 5% significance level (two-sided) using js-STAR XR (release 1.4.0 j).

## Results

### Experiment 1. Effect of low pH on cytotoxicity and MN induction

**Fig. 3:**
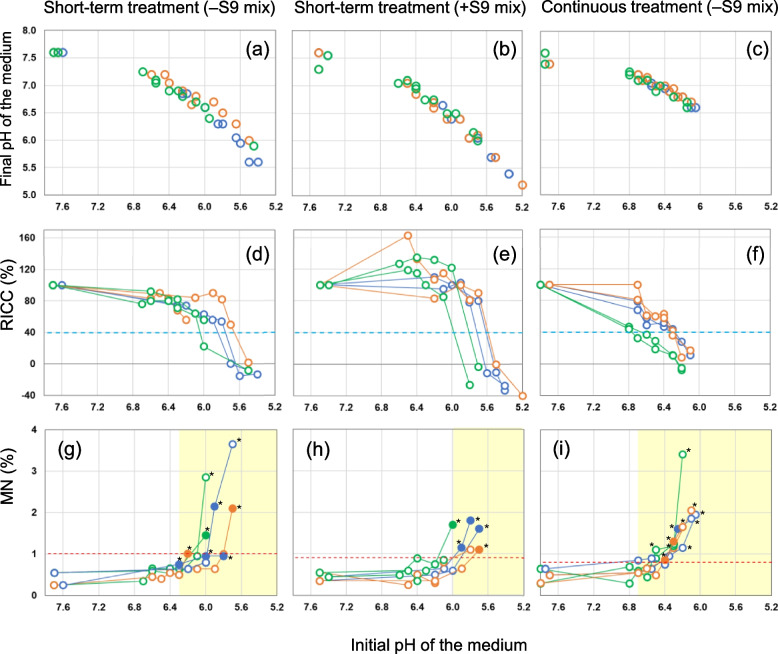
Effect of low pH on cytotoxicity and MN induction. The results of the two experiments were plotted. **a**–**c** Final pH of the medium; **d**–**f** RICC (%); **g**–**i** MN frequencies (%).  , Acetic acid;  , Lactic acid;  , Formic acid;  , RICC toxic level (40%);  , Upper limit of historical negative control range.  , Values in groups with significant increases in MN frequencies (*p* < 0.05, Fisher's exact test) and RICC ≥ 40%. *, Significantly increased values in MN frequencies (*p* < 0.05, Fisher's exact test). Statistical analysis was performed between the treated and the corresponding negative control groups

When the initial pH of the medium decreased to below 6.8, the final pH (at 3 and 24 h in the short-term and continuous treatments, respectively) did not increase to that (7.4–7.8) of those in the untreated groups (Fig. 3a–c). The results of the two experiments were plotted.

The changes in the RICC with decreasing initial pH are presented in Fig. 3d–f, and the results of the two experiments were plotted. Although there was variation between the experiments, RICC decreased with the decrease in the pH of the medium in both short-term and continuous treatments. In the short-term treatment, RICC decreased sharply at a pH of approximately 6.0 and fell below 40%. MN analyses could not be performed when RICC was ≤ 0%.

Figure 3g–i illustrate the changes in MN frequencies with the decrease in initial pH. The results of the two experiments were plotted. In the yellowish area of the pH range, the MN frequencies in the treated groups increased significantly compared with those in the untreated group, and increased above the upper limit of the historical negative control range in our laboratory. The pH range in which these values were distributed was below pH 6.3 and 6.0 in the absence and presence of S9 mix in the short-term treatment, respectively, and below 6.7 in the continuous treatment. Significant increases in MN frequencies were observed in almost all groups where the RICC was < 40% and also in the groups where the RICC was ≥ 40% (solid circles in Fig. 3g–i) despite low pH, particularly in the short-term treatment.

The above results can be considered false positives because the genotoxicity of these organic acids is negative.

### Experiment 2 Investigation of applicable antibiotic concentration ranges for recovery culture

**Fig. 4:**
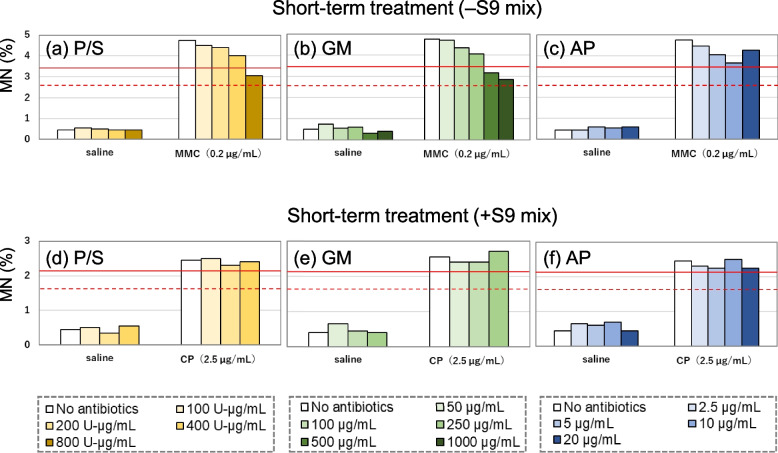
Effect of various antibiotic concentrations on MN frequencies in TK6 cells. Cells were treated with negative or positive controls for 3 h in the absence (**a**–**c**) or presence (**d**–**f**) of S9 mix, followed by recovery culturing for 21 h using media supplemented with various concentrations of P/S (a and d), GM (b and e), or AP (c and f). MMC, 0.2 µg/mL; CP, 2.5 µg/mL;  , Mean of the historical positive control value;  , Lower limit of historical positive control range

Figure 4a–c and d–f present the MN frequencies of the negative and positive control groups in short-term treatment with and without S9 mix, respectively, when antibiotic-free or antibiotic-supplemented medium was used during the recovery period.

The MN frequencies in the negative control groups were similar, irrespective of antibiotic supplementation and were within the historical negative control range.

The MN frequencies in the positive control groups were above the lower limit of the historical positive control ranges at all antibiotic concentrations in the absence of S9 mix. However, the values decreased with the increasing P/S and GM concentrations to below the mean of the historical positive control data at 800 U-µg/mL P/S and 500 and 1,000 µg/mL GM. Thus, in the presence of S9 mix, 400 U-µg/mL P/S, 250 µg/mL GM, and 20 µg/mL AP were the maximum concentrations used in this study. MN frequencies in the positive control groups were above the mean of the previous positive control data at all antibiotic concentrations used in the presence of S9 mix.

Therefore, the antibiotic concentrations that did not affect MN induction in TK6 cells were determined to be 100–400 U-µg/mL for P/S, 50–250 µg/mL for GM, and 2.5–20 µg/mL for AP.

### Experiment 3. Verification of the effect of antibiotics in recovery cultures on the results of the MNT using sample A containing live lactic acid-producing bacteria

**Fig. 5:**
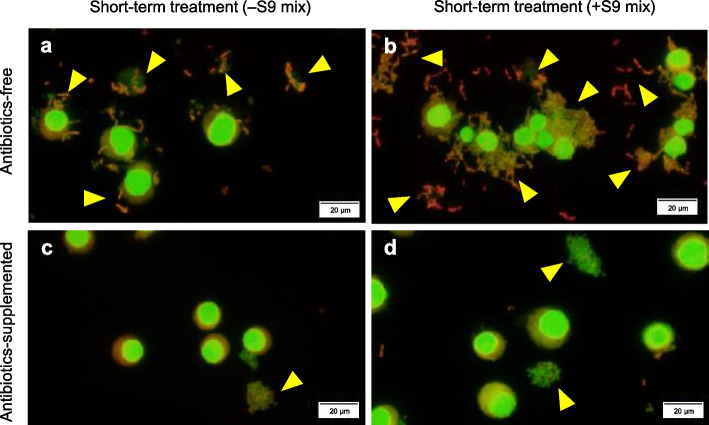
Representative images of specimens in sample A groups. Cells were treated with sample A for 3 h in the absence (**a**, **c**) or presence (**b**, **d**) of S9 mix, followed by recovery culturing for 21 h. The media used in the recovery cultures were as follows: **a**, **b**, antibiotic-free medium; **c**, **d**, antibiotic-supplemented medium (100 U-µg/mL P/S). The arrowheads indicate the remaining bacteria derived from sample A

MN tests were performed using antibiotic-free and -supplemented media (P/S 100 and 400 U-µg/mL or GM 50 µg/mL) for recovery culturing after 3 h of treatment with sample A, saline, or positive controls in the absence or presence of S9 mix. Table 1 presents the final pH of the medium (at the end of the recovery period) and RICC in each group. Decreases in both values were observed only in the groups treated with antibiotic-free medium during the treatment and recovery periods, and marked bacterial growth was observed at the end of the recovery period in these groups. In the presence of S9 mix, MN analysis was impossible because of the large number of bacteria overlapping the TK6 cells in the specimens (Fig. 5a and b).

**Table 1:** Antibiotic effect on the final pH and cytotoxicity of TK6 cells treated with sample A

Treatment condition				Final pH of the medium	RICC (%)
S9 mix	Test substance (final conc.)	Antibiotics ^1)^			
–	Saline	–	0	7.4	100
	Sample A (10 µL/mL)	–	0	6.9	61
		P/S	100 U-µg/mL	7.4	102
			400 U-µg/mL	7.4	100
		GM	50 µg/mL	7.4	100
	MMC (0.2 µg/mL)	–	0	7.5	50
+	Saline	–	0	7.4	100
	Sample A (10 µL/mL)	–	0	6.9	29
		P/S	100 U-µg/mL	7.4	108
			400 U-µg/mL	7.4	86
		GM	50 µg/mL	7.4	94
	CP (2.5 µg/mL)	–	0	7.5	67

1) Final concentration of the antibiotics in the medium

*MMC* Mitomycin C, *CP* Cyclophosphamide monohydrate, *P/S* Penicillin/streptomycin, *GM* Gentamicin sulfate, *AP* Amphotericin B

**Fig. 6:**
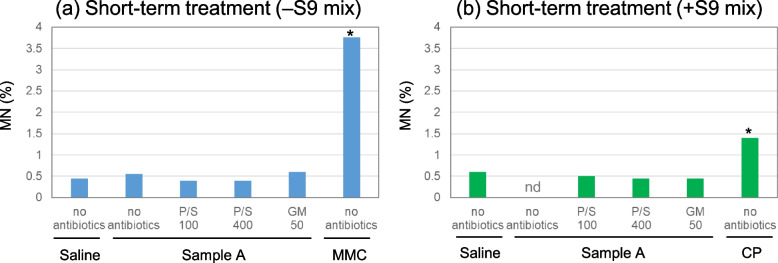
Results of the MNT of sample A using media with or without antibiotics. Cells were treated with sample A for 3 h in the absence (**a**) or presence (**b**) of S9 mix, followed by recovery culturing for 21 h using media supplemented with 100 and 400 U-µg/mL PS, or 50 µg/mL GM. nd, unable to observe due to aggregated bacteria; MMC, 0.2 µg/mL; CP, 2.5 µg/mL. *p < 0.05, compared with the negative control (saline group; Fisher’s exact test)

Contrastingly, bacterial growth was considerably suppressed in the sample A groups using the antibiotic-supplemented medium and did not interfere with MN analyses (Fig. 5c and d). The pH values were identical to those in the saline group, and the RICC was ≥ 80% for all antibiotic concentrations (Table 1). There was no increase in the MN frequency (Fig. 6a and b).

Herein, the MN frequency in the negative control group (saline group) was within the historical negative control range in our laboratory. The MN frequencies in the positive control groups (MMC and CP) were significantly higher than those in the saline group. Therefore, this study was conducted appropriately.

## Discussion

When the genotoxicity of a test substance containing probiotic candidates, such as lactic acid-producing bacteria, is assessed through MNT using cultured cells, bacterial growth in the test medium may reduce the pH of the medium, inhibit cell growth, and induce false positive results. In the present study, we examined the effect of low pH on TK6 MNTs by treating them with non-genotoxic organic acids (acetic, lactic, or formic acid), which are typical bacterial metabolites. As the pH of the media decreased with the addition of organic acids, false positive responses in MN frequencies (significant increases compared with the concurrent negative controls and/or increases above the upper limit of the historical negative control range) were observed. The pH range yielding false positives was below 6.3 and 6.0 in the absence and presence of S9 mix (short-term treatment), respectively, and below 6.7 in continuous treatment. The false positive-inducing low pH value was higher in the continuous treatment than that in the short-term treatment, probably due to the longer duration of the treatment at low pH [7]. Low pH can induce chromosomal aberrations or micronuclei in cultured mammalian cells [1, 2], and the clastogenic response to low pH differs greatly among cell lines [2, 5–8]. The TK6 cell line is widely used for in vitro MNT, and is recommended in OECD TG 487 [4]; however, the precise pH values that cause false positives in in vitro MNT have not been described in the guideline. Therefore, the false positive-inducing pH range indicated in this study will be helpful in interpreting the biological relevance of the TK6 cell MNT results.

OECD TG 487 [4] specifies that the highest concentration should aim to achieve 55 ± 5% cytotoxicity using the recommended cytotoxicity parameters to avoid false positive results due to excessive cytotoxicity. When RICC is used as the parameter, < 40% of RICC corresponds to the excessive cytotoxicity level. Herein, false positive responses in MN frequencies were observed in almost all groups where the RICC was < 40%, indicating excessive cytotoxicity. Moreover, false positive responses were also observed in the groups where the RICC was ≥ 40% despite low pH, indicating low pH itself induced micronucleated cells. The induction of chromosomal aberrations by low pH may involve inhibition of DNA or protein synthesis, DNA destabilization, and inhibition of DNA repair pathways [2, 8, 13, 14].

To examine the effect of antibiotic supplementation on bacterial growth suppression in recovery cultures post-treatment of the live bacteria-containing test substance, the maximum applicable concentration of the three antibiotics was investigated. These antibiotics did not affect cell proliferation or MN frequencies in the negative and positive control groups at concentrations up to 400 U-µg/mL P/S, 250 µg/mL GM, and 20 µg/mL AP. These values will guide the use of antibiotics; however, the optimal antibiotic type and concentration depends on the type of live bacteria present in the test substance. It is unclear why the MN frequencies in the MMC groups treated with 800 U-µg/mL P/S and 500 and 1,000 µg/mL GM were lower than those in the groups treated with lower concentrations of antibiotics. However, these phenomena could be attributed to the potential effects of antibiotics, such as altering gene expression, cell proliferation, and cell cycle [15–17].

Finally, we conducted a TK6 cell MNT using sample A as a test substance containing live lactic acid-producing bacteria to verify the effect of antibiotic supplementation on recovery cultures. Compared to the use of antibiotic-free medium, using medium supplemented with antibiotics (100 and 400 U-µg/mL P/S or 50 µg/mL GM) during the recovery period suppressed bacterial growth, resulting in no decrease in the pH of the medium or no cytotoxicity. MN analysis could be performed in all groups using antibiotic-supplemented media, whereas it could not be performed in the group using antibiotic-free medium in the presence of S9 mix. Incidentally, sample A could be evaluated at 10 µL/mL using antibiotic-supplemented media for recovery cultures in short-term treatment; however, in continuous treatment using antibiotic-free media, a lower dose (4 µL/mL) was the maximum as the final pH and RICC were 6.6 and approximately 30%, respectively (data not shown), suggesting that antibiotic supplementation for recovery cultures is a useful method for studying the influence of live bacteria-containing test substances (up to higher doses) on TK6 cells, compared to that of the media without antibiotics.

From these results, we believe that this method could be a useful approach to evaluate the clastogenicity of test substances containing probiotic candidates using in vitro MNT, but further studies (e.g., validation with various probiotics and the other approaches, such as a test substance treatment via cell culture insert) are also needed.

## Conclusion

This study revealed the low pH ranges yielding false positives in the TK6 cell MNT under short-term and continuous treatment conditions. These values will serve as references for interpreting the biological relevance of results. Under short-term treatment, optimal antibiotic supplementation in recovery cultures suppressed bacterial growth in the test substance and prevented the decrease in pH that could yield false positives. This approach might be useful for evaluating the genotoxicity of test substances containing probiotic candidates using the MNT.

## Data Availability

.All data generated in this study are included in this published article.

## Abbreviations

MN: Micronucleus
MNT: Micronucleus test
RICC: Relative increase in cell count
P/S: Penicillin/streptomycin
GM: Gentamicin sulfate
AP: Amphotericin B
MMC: Mitomycin C
CP: Cyclophosphamide monohydrate

## References

[1] Morita T, Watanabe Y, Takeda K, Okumura K. Effects of pH in the *in vitro* chromosomal aberration test. Mutat Res. 1989;225:55–60. 10.1016/0165-7992(89)90033-x. 2913491 10.1016/0165-7992(89)90033-x

[2] Meintieres S, Marzin D. Apoptosis may contribute to false-positive results in the *in vitro* micronucleus test performed in extreme osmolality, ionic strength, and pH conditions. Mutat Res. 2004;560:101–18. 10.1016/j.mrgentox.2004.02.003. 15157649 10.1016/j.mrgentox.2004.02.003

[3] OECD. Test no. 473, in vitro mammalian chromosomal aberration test. OECD guideline for the testing of chemicals. Paris: OECD; 2016.

[4] OECD. Test no. 487, in vitro mammalian cell micronucleus test. OECD guideline for the testing of chemicals. Paris: OECD; 2016.

[5] Sinha AK, Gallapudi BB, Linscombe VA, McClintock ML. Utilization of rat lymphocytes for the *in vitro* chromosomal aberration assay. Mutagenesis. 1989;4:147–53. 10.1093/mutage/4.2.147. 2659926 10.1093/mutage/4.2.147

[6] Kalweit S, Nowak C, Obe G. Hypotonic treatment leads to chromosomal aberrations but not to sister-chromatid exchanges in human lymphocytes. Mutat Res. 1990;4:147–53. 10.1016/0165-7992(90)90017-e. 10.1016/0165-7992(90)90017-e 2392128

[7] Morita T, Takeda K, Okumura K. Evaluation of clastogenicity of formic acid, acetic acid and lactic acid on cultured mammalian cells. Mutat Res. 1990;240:195–202. 10.1016/0165-1218(90)90058-A. 2314411 10.1016/0165-1218(90)90058-a

[8] Morita T, Nagaki T, Fukuda I, Okumura K. Clastogenicity of low pH to various cultured mammalian cells. Mutat Res. 1992;268:297–305. 10.1016/0027-5107(92)90235-T. 1379335 10.1016/0027-5107(92)90235-t

[9] Sofuni T. Data book of chromosomal aberration test in vitro. Revised ed. Saitama: Life-science Information Center; 1999. p.28–29, p.300.

[10] Kawanishi M, Hisatomi Y, Oda Y, Shimohara C, Tsunematsu Y, Sato M, et al. In vitro genotoxicity analyses of colibactin-producing *Escherichia coli* isolated from a Japanese patient with colorectal cancer. J Toxicol Sci. 2019;44:871–6. 10.2131/jts.44.871. 31813906 10.2131/jts.44.871

[11] ATCC. Animal cell culture guide. https://www.atcc.org/resources/culture-guides/animal-cell-culture-guide#Complete. Accessed 30 Oct 2024.

[12] Perlman D. Use of antibiotics in cell culture media. In: Jacoby WB, Pastan IH, editors. Cell culture: methods in enzymology, vol. 58. New York: Academic Press; 1979. p.110–116. 10.1016/s0076-6879(79)58128-2 423753

[13] Chambard JC, Poiyssegur J. Intracellular pH controls growth factor-induced ribosomal protein S6 phosphorylation and protein synthesis in the G0–G1 transition of fibroblasts. Exp Cell Res. 1986;164:282–94. 10.1016/0014-4827(86)90029-7. 3011468 10.1016/0014-4827(86)90029-7

[14] Fukuda T, Komaki Y, Mori Y, Ibuki Y. Low extracellular pH inhibits nucleotide excision repair. Mutat Res. 2021;867: 503374. 10.1016/j.mrgentox.2021.503374. 10.1016/j.mrgentox.2021.503374 34266626

[15] Ryu AH, Eckalbar WL, Kreimer A, Yosef N, Ahituv N. Use antibiotics in cell culture with caution: genome-wide identification of antibiotic-induced changes in gene expression and regulation. Sci Rep. 2017;7:7533. 10.1038/s41598-017-07757-w. 28790348 10.1038/s41598-017-07757-w PMC5548911

[16] Neftel KA, Hubscher U. Effects of beta-lactam antibiotics on proliferating eucaryotic cells. Antimicrob Agents Chemother. 1987;49:29–56. 10.1128/aac.31.11.1657. 10.1128/aac.31.11.1657 PMC175015 3324959

[17] Fischer AB. Gentamicin as a bactericidal antibiotic in tissue culture. Med Microbiol Immunol. 1975;161:23–39. 10.1007/BF02120767. 236490 10.1007/BF02120767

